# A new generation of patient-reported outcome measures with large language models

**DOI:** 10.1186/s41687-025-00867-4

**Published:** 2025-03-24

**Authors:** Jan Henrik Terheyden, Maren Pielka, Tobias Schneider, Frank G. Holz, Rafet Sifa

**Affiliations:** 1 University Hospital Bonn, Department of Ophthalmology, Venusberg-Campus 1, 53127 Bonn, Germany; 2 Bonn-Aachen International Center for Information Technology, University of Bonn, Friedrich- Hirzebruch-Allee 6, 53115 Bonn, Germany; 3 Media Engineering Department, Fraunhofer IAIS, Schloss Birlinghoven 1, 53757 Sankt Augustin, Germany

**Keywords:** Patient-reported outcome measures, Large Language models, Generative artificial intelligence, Digital medicine

## Abstract

**Background:**

Patient-reported outcome measures (PROMs) are cornerstones of patient-centered clinical medicine and reflect patients’ abilities, difficulties, perceptions and behaviors. The highly structured questionnaire format of PROMs currently limits their real-world validity and acceptability to patients, which becomes increasingly relevant with the high clinical interest in PROM data. In this short commentary, we aim to demonstrate the potential use of large language models (LLMs) in the context of PROM data collection and interpretation.

**Main body:**

The popularization of LLMs enables the development of a new generation of PROMs generated and administered through digital technology that interact with patients and score their responses in real time based on artificial intelligence. LLM-PROMs will need to be developed with multi-stakeholder input and careful validation against established PROMs. LLM-PROMs could complement traditional PROMs particularly in real-world clinical applications.

**Conclusion:**

LLM-PROMs could allow quantifying patient-relevant dimensions based on less structured contents and foster the use of patient-reported data in digital, clinical applications of PROMs.

## Background

Patient-reported outcome measures (PROMs) are central to patient-centered clinical medicine and assess health domains such as health-related quality of life, symptoms and health behaviors, allowing practitioners to tailor treatment approaches to patient needs [1–3]. PROMs are increasingly used as trial endpoints, in quality assessment of healthcare programs and during routine care, as they are ideal candidates for obtaining health information outside clinical settings, e.g. during remote monitoring of chronic conditions [1–3]. However, the high degree of standardization necessary for the development of reliable PROM instruments implies that patients are asked to complete structured, inflexible questionnaires [4, 5]. The advent of large language models (LLMs) is one of the breakthroughs in current artificial intelligence (AI) technology and can help to transform healthcare at a large scale [6, 7]. Popularized with the software ChatGPT (OpenAI, San Francisco, CA) [8], a plurality of public and private LLMs have been proposed for use in healthcare settings, ranging from diagnostic and therapeutic approaches to research and medical training purposes [9]. Despite this, individualizing and measuring quantitative, patient-reported outcome domains through LLMs has not yet gained attention. Thus, we aim to demonstrate the potential use of large language models (LLMs) in the context of PROM data collection and interpretation in this short commentary. The newly introduced term LLM-PROM describes a hybrid system generating individualized, open-ended PROM items and numerically interpreting patient responses.

## Main text

Since standardized patient questionnaires were first used to measure health outcomes in the 1960s, the understanding of PROM design, validation, application, and interpretation has made significant progress [1]. PROM contents are developed by clinicians, researchers and policymakers with qualitative input from patients, medical experts and the medical literature [1, 10]. Based on this, PROMs can be applied to a broad range of people, including the general population and those with specific medical conditions (generic and condition-specific measures) [1]. The highly structured format of PROMs including items and response options is based on these qualitative development steps. This structured format is widely accepted in the pharmacoregulation context in which PROMs were first developed and are commonly used. One major advantage of this structured format is to ensure that the items and subscales are interpreted in the same way across different populations so that treatment and time changes can be assessed at an inter-individual level. For clinical trials, ensuring comparable responses and broad item coverage remains essential for assessing the patient relevance of new treatments. Requirements of PROM use in routine healthcare differ from this and administering a complex questionnaire to patients’ needs to be justified by feasibility and added benefit. PROMs that implement a higher degree of personalization in sub-populations (e.g. in the context of individually relevant activities of daily living, comprehensibility as per individual language level) promise to generate an increasing impact in the context of clinical care. However, PROMs are prone to missing data [11], which can severely impact the efficacy and effectiveness of outcome measurement in health care programs.

Significant efforts are put into developing short forms of PROMs that may be faster to administer during routine care but can also lose precision compared to the full-length instrument. In the context of latent trait models, item banking has been introduced to make the assessments better targeted. Computer adaptive testing (CAT) deconstructs and individualizes PROMs into combinations of single items [1]. While this approach may reduce the complexity of an assessment, it may not necessarily capture all content domains relevant to an individual patient. As opposed to PROMs, patient-generated outcome measures (PGOMs) summarize individualized questionnaire tools that are developed with an individual person with a high effort to create one measure per individual patient, targeting their respective needs [12–14]. PGOMs have been suggested as a complement to PROMs or to support treatment decisions but not as a replacement of existing instruments for real-world healthcare [13]. Overall, the foundational principles underlying the definition of a PROM have remained largely unchanged from their original conceptualization, which could limit their application in real-world care settings.

Natural language processing (NLP) describes making natural languages (as opposed to software code) readable and computable by machines [15]. NLP is the foundation for the development of LLMs, i.e. AI models that were trained to generate and interpret human language [16]. While the prognostic value of clinician-reports can be limited, patient-reports provide a rich information source about various domains regarding symptoms, quality of life and health behaviors [17]. The quantification of patients’ perspectives currently requires rigid instruments that are highly structured. The resulting assessment could potentially be limited by individual respondents’ motivations and backgrounds (e.g. educational and cultural backgrounds not covered in validation studies) [18]. One of the main capabilities of AI is the interpretation of unstructured data. Using LLMs and NLP to generate and interpret personalized patient interactions could lead to a new generation of PROMs.

LLM-PROMs are LLM-based psychometric measurement instruments capturing patient-reported outcome data. LLM-PROMs combine two core functionalities: Item generation and patient-report interpretation, which are both conducted by algorithms (Table 1).

**Table 1:** Conceptualization of large language model - patient-reported outcome measures

PROM component	Equivalent in LLM-PROM	
Item	Algorithmically generated, open-ended question content valid in the context of a given medical condition that is directed to a patient	
Response (per response scale)	Patient’s reply to a given LLM-PROM item validated for the given use scenario	

LLM, large language model; PROM, patient-reported outcome measure

**Fig. 1:**
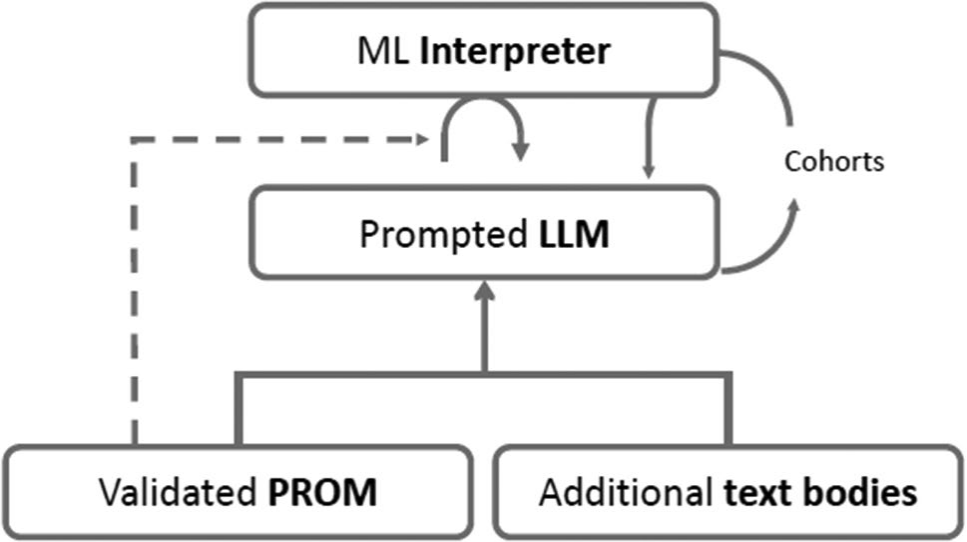
Development framework of a large-language model patient-reported outcome measure (LLM-PROM). LLM, large language model; ML, machine learning; PROM, patient-reported outcome measure

Therefore, LLM-PROMs combine individualized open-ended items (e.g. text messages, audio material read to the patient) with the interpretation of responses by a machine learning algorithm trained to derive quantitative metrics out of its responses (Fig. 1). The suggested framework of LLM-PROMs is shown in Fig. 1 and consists of three layers. The foundation of LLM-PROMs are text contents, which could be derived e.g. from existing PROMs or qualitative datasets of patient or expert interviews, or focus group discussions. The second layer consists of the LLM which is supplied with a pre-defined input (prompt) that captures essential aspects of the intended contents of the PROM. Examples of contexts captured are in line with existing PROMs and may include health-related quality of life, symptom burden or health behaviors. The result of this becomes the interaction between a patient and a digital bot (e.g. chat bot, voice bot) with a focus on the concept of the LLM-PROM (e.g. health-related quality of life). In contrast to conventional PROMs, the linguistic context of LLM-PROM items can be dynamically adapted to the respondent during the process, e.g. with regards to preferences, language use or cultural background. LLM-PROMs share similarities with existing adaptive PROMs (e.g. CAT-based systems) but are based on open-ended items and can introduce items not covered by an existing item bank. Based on the between a patient and an LLM-PROM, a machine learning algorithm (“interpreter”) infers quantitative metrics capturing the very aspects for which structured questionnaire responses are a current requirement.

Like PROMs, LLM-PROMs must be rigorously tested for objectivity, reliability, validity, and responsiveness before clinical use. In this context, existing PROMs will likely need to remain the gold standard for establishing convergent validity and LLM-PROMs will need to prove predictive validity and responsiveness prior to clinical use. Furthermore, the administration burden of LLM-PROMs will need to be evaluated precisely before they can be implemented in routine health care delivery. While AI approaches have been used to score data from existing PROMs [19], the developments in LLMs seen today create the opportunity to introduce the dimension of personalized medicine into PROM assessment which could be useful for guiding treatment decisions, capturing symptoms and care needs as well as adherence and safety monitoring. Thus, it could become possible to obtain a new category of quantitative metrics from written and spoken natural language. This might not only reduce the administration burden of existing PROMs but contribute to the overall goal of a more patient-centered healthcare through LLM-PROMs.

The development of LLM-PROMs requires scientifically robust methods to ensure the content validity of the instruments. The importance of this is highlighted by the fact that the phrasing of items used in LLM-PROMs is not driven by work with patients directly but originates from prompts to the large language model. However, the opportunity to include large bodies of text into the back-end of the LLM-PROM framework (Fig. 1) may hypothetically even increase the patient-relevance of LLM-PROMs over traditional PROMs. Biases of LLM-PROMs will be an important topic of research. For instance, the sentiment (“mood”) in patient responses may be affected by priming effects. Furthermore, biases towards sociodemographic groups (e.g., based on sex, age, ethnicity) [20] will be important topics to consider during the development of LLM-PROMs. We therefore propose using heterogeneous training sets for AI models. They should particularly reflect the populations in which the LLM-PROMs are intended to be used (e.g., in terms of sex, age, ethnicity). Specific types of biases that require to be addressed include minority bias, missing data bias and informed mistrust [21, 22]. Furthermore, we suggest to develop a safety filter network to correct for these biases. Such a filter mechanism could be a separate application that detects inappropriate LLM-PROM items before these are displayed to the respondent. Currently, the sizes of data sets required to effectively capture PROM variables with machine learning algorithms are largely unclear and it may be troublesome to train machine learning interpreters to cover rare conditions since large databases will be required. Further research will be needed to stratify the types of cohorts needed to rigorously validate an LLM-PROM and ensure its real-life validity for healthcare applications.

Current digital applications of PROMs mostly reflect their pen and paper equivalents, while the term digital transition could imply using digitization to rethink existing processes from the start. The integration of LLM-PROMs into digital chat bots could allow novel, personalized and patient-centered models of telemedicine by facilitating the collection of PROM data in existing care pathways. This may not only hold true for remote monitoring applications in chronic conditions and postoperative care but further impact digital treatment regimens in behavioral health. While applications of LLM-PROMs in clinical and research contexts could be implemented in the near future, their use in clinical trials may remain challenging as no structured regulatory pathways for use of AI in the context of PROMs exist yet. For the integration of LLM-PROMs into clinical care pathways, their interaction with electronic health records will need to be demonstrated, posing potential implementation challenges.

A large language model is prompted based on an existing, validated patient-reported outcome measure (source PROM) and additional text bodies. A machine learning algorithm is trained to estimate the LLM-PROM score based on individuals’ free-text responses based on a user interaction and source PROM scores.

LLM-PROMs could hypothetically alter the adoption of personalized medicine during outcome assessment since individual contributors to e.g. health-related quality of life or health behaviors could be targeted to the individual patient. This might be beneficial from a content validity perspective, pending empirical validation. Furthermore, LLM-PROMs could potentially improve the comprehensibility of PROM assessment given the inter-individual differences in language levels and health literacy. Lastly, the use of digital technology with LLM-PROMs promises to hypothetically reduce the loss of PROM data during routine healthcare supervision by addressing patient needs more individually than traditional, highly structured PROMs. Overall, LLM-PROMs remain in the early stages of development, and further scientific evaluation is required to determine their validity and effectiveness.

In the early stages of a new field, a conceptualization framework of LLM-PROMs will be a key requirement. Similarly to PROM development guidelines, scientific standards should direct the strategical development of LLM-PROMs for the healthcare, psychometrics and AI communities. First and foremost, knowledge from existing PROMs needs to be used to develop application-specific vocabularies and safety control mechanisms since the use of LLM-PROMs implies that AI algorithms communicate directly with patients. Similarly to the use of AI in medical imaging, using LLMs to measure patient-reported data will generate a diagnostic black box less available for external evaluation and auditing than conventional and model-based approaches. This issue is under debate for most use cases of AI in healthcare and no final conclusions can be drawn at this point, since broader societal involvement is needed. Integrating patient organizations at the core of these developments will be a key factor ensuring that LLM-PROMs actually foster patient-centered care.

## Conclusions

Generative AI and LLMs hold the potential for the development of a novel type of PROMs. Ensuring content validity, appropriateness and psychometric robustness of these instruments will be key enablers of their success. Healthcare providers, researchers, patients and organizations will need to align on a conceptual framework for the development of LLM-PROMs at an early stage.

## Data Availability

Not applicable.

## Abbreviations

AI: Artificial intelligence
LLM: Large language model
NLP: Natural language processing
PROMs: Patient-reported outcome measures
